# Novel Nanocomposite Materials for Advanced Li-Ion Rechargeable Batteries

**DOI:** 10.3390/ma2031205

**Published:** 2009-09-03

**Authors:** Chuan Cai, Ying Wang

**Affiliations:** Department of Mechanical Engineering, Louisiana State University, Baton Rouge, LA 70803, USA

**Keywords:** lithium-ion battery, nanocomposites, cathode, anode

## Abstract

Nanostructured materials lie at the heart of fundamental advances in efficient energy storage and/or conversion, in which surface processes and transport kinetics play determining roles. Nanocomposite materials will have a further enhancement in properties compared to their constituent phases. This Review describes some recent developments of nanocomposite materials for high-performance Li-ion rechargeable batteries, including carbon-oxide nanocomposites, polymer-oxide nanocomposites, metal-oxide nanocomposites, and silicon-based nanocomposites, etc. The major goal of this Review is to highlight some new progress in using these nanocomposite materials as electrodes to develop Li-ion rechargeable batteries with high energy density, high rate capability, and excellent cycling stability.

## Contents

1. **Introduction**
2. **Nanosized Coatings on Cathode Materials**
  2.1. Nanosized coatings on lithium transition metal oxides
  2.2. Nanosized coatings on lithium phosphates
3. **Nanostructured Composites as Cathode Materials**
  3.1. Nanostructured carbon-oxide composites
  3.2. Nanostructured polymer-oxide composites
  3.2. Nanostructured metal-oxide composites and other nanocomposites
4. **Nanostructured Composite as Anode Materials**
  4.1. Nanostructured silicon-carbon composites
  4.2. Nanostructured tin-carbon composites
  4.3. Nanostructured tin oxide-carbon composites
  4.4. Nanostructured transition metal oxide-carbon composites
  4.5. Other nanostructured composites
5. **Concluding Remarks**

## 1. Introduction

Recent increases in demand for oil, associated price increases, and environmental issues are continuing to exert pressure on an already stretched world energy infrastructure. One alternative energy/power source under serious consideration is electrochemical energy production, as long as this energy consumption is designed to be more sustainable and more environmentally benign. The lithium-ion battery is the representative system for such electrochemical energy storage and conversion. At present lithium-ion batteries are efficient, light-weight and rechargeable power sources for consumer electronics, such as laptop computers, digital cameras and cellular phones [1]. Moreover, they have been intensively studied for use as power supplies of electric vehicles (EVs) and hybrid electric vehicles (HEVs). High energy and high power densities are required for such devices. Lithium-ion batteries are attractive power-storage devices owning to their high energy density [2]. However, their power density is relatively low because of a large polarization at high charging-discharging rates. This polarization is caused by slow lithium diffusion in the active material and increases in the resistance of the electrolyte when the charging-discharging rate is increased. To solve these problems, it is important to design and fabricate nanostructured electrode materials that provide high surface area and short diffusion paths for ionic transport and electronic conduction.

**Figure 1:**
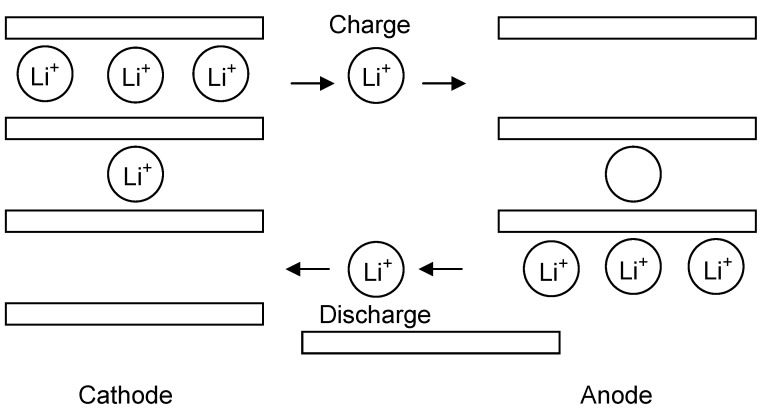
Schematic illustration of a lithium-ion battery.

The principal concept of lithium-ion batteries is illustrated in Figure 1. A combination of a negative lithium intercalation material (anode) with another lithium intercalation material (cathode) having a more positive redox potential gives a Li-ion transfer cell. Anode and cathode are separated by the electrolyte which is an electronic insulator but a Li-ion conductor. Upon charging, lithium ions are released by the cathode and intercalated at the anode. When the cell is discharged, lithium ions are extracted by the cathode and inserted into the anode. Electrode materials must fulfill three fundamental requirements to reach the goal of a high specific energy and energy density: (1) a high specific charge and charge density, *i.e.*, a high number of available charge carriers per mass and volume unit of the material; (2) a high cell voltage, resulting from a high (cathode) and low (anode) standard redox potential of the respective electrode redox reaction; and (3) a high reversibility of electrochemical reactions at both cathodes and anodes to maintain the specific charge for hundreds of charge/discharge cycles.

Nanomaterials offer the unusual mechanical, electrical and optical properties endowed by confining the dimensions of such materials. The overall behavior of nanomaterials exhibit combinations of bulk and surface properties [3]. The reports on the processing, properties and applications of nanomaterials are rapidly appearing on daily basis. It is clear that nanostructured electrodes offer improved energy storage capacity and charge/discharge kinetics, as well as better cyclic stabilities due to their huge surface area for Faradaic reaction, and short distance for mass and charge diffusion, as well as the added freedom for volume change during the Li-ion intercalation/deintercalation process.

On the other hand, nanostructured electrodes also introduce new challenges. For example, nanoparticles of LiCoO_2_ and LiNiO_2_ can react with the electrolyte; nanoparticles of LiMn_2_O_4_ can dissolve in the electrolyte during cycling. The problems of poor electric conductivity of some cathode materials (e.g., LiFePO_4_, MnO_2_, SnO_2_, TiO_2_) and the issues of drastic volume change of some anode materials (e.g., Si, Sn) during cycling cannot be solved by only downsizing the materials to the nanoscale. In addition, nanoparticles of electrode materials tend to agglomerate, resulting in less surface area and reduced electrochemical activity. One solution to these problems is to employ nanocomposites as electrode materials, since nanocomposite materials show even better properties than the constituent components. Typically, composite electrode materials can be prepared by depositing surface coatings on the active materials, dispersing active materials in the host matrices, or adding inactive materials into the active materials. The surface coating on the electrode material serves as a protection layer to hinder the undesirable side reactions with the electrolyte or the dissolution of the active material. Dispersing the electrode material in the host matrix will introduce more freedom for volume change during cycling and hinder the agglomeration of the electrode material. Adding nanosized conductive materials into the electrode material can facilitate the diffusion of Li ions or improve the electronic conductivity and morphological stability.

This article aims to provide a useful survey of recent progress on synthesis and characterizations of nanocomposite electrode materials for lithium-ion batteries. We will start with discussions on nanosized coating on some cathode materials such as lithium transition metal oxides and lithium phosphates in Section 2. Section 3 and Section 4 cover the nanocomposite cathode materials and nanocompsite anode materials, respectively. Each section includes a variety of nanocomposite materials such as carbon-oxide nanocomposites, polymer-oxide nanocomposites, and metal-oxide nanocomposites as cathode materials, and silicon-based, tin-based, and transition metal oxides-based nanocomposites as anode materials.

## 2. Nanosized Coatings on Cathode Materials

### 2.1. Nanosized coatings on lithium transition metal oxides

It should be noted that the nanoparticulate forms of lithium transition metal oxides such as LiCoO_2_, LiNiO_2_, or their solid solutions, can react with the electrolyte and cause safety problems. In the case of LiMn_2_O_4_, the use of nanoparticles causes undesirable dissolution of Mn. Better stability can be achieved by coating the electrode materials with a nanosized stabilizing surface layer that alleviates these problems.

LiCoO_2_ is the most popular among the possible cathode materials owing to the convenience and simplicity of preparation. This material can be easily synthesized using both solid-state and chemical approaches [4,5]. The Li_*x*_CoO_2_ exhibits excellent cyclability at room temperature for 1 > *x* > 0.5. Therefore, the specific capacity of the material is limited to the range of 137 to 140 mAh/g, although the theoretical capacity of LiCoO_2_ is 273 mAh/g [6]. On the other hand, Li_*x*_CoO_2_ is very expensive and highly toxic.

As for LiCoO_2_, coatings of carbon, various phosphates and oxides have been studied and significant improvements in capacity retention have been demonstrated. Carbon-coating can enhance the structural stability and electrical conductivity of LiCoO_2_ [7], but LiCoO_2_ may be reduced to CoO or Co_3_O_4_ by carbon [8]. Nanosized carbon coating has been deposited on LiCoO_2_ by milling with sucrose followed by calcination in air [9]. The obtained composite cathode shows a higher capacity than the pristine LiCoO_2_, which is ascribed to the reduced charge transfer resistance and the faster Li-ion diffusion.

**Figure 2:**
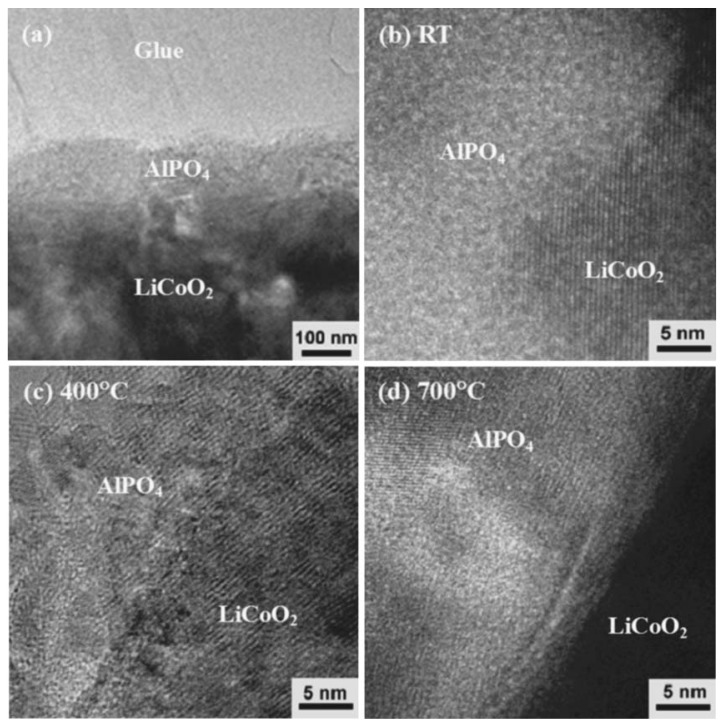
(a) Cross-sectional TEM images of AlPO_4_-coated LiCoO_2_. A ~100 nm thick AlPO_4_ continuous layer is coated on the LiCoO_2_. High resolution images of the AlPO4-coated LiCoO_2_ at (b) room temperature, (c) 400 ºC, and (d) 700 ºC. Adapted from [13]. Copyright 2006 The Electrochemical Society.

Apart from carbon coating, various MPO_4_ (M = Al, Fe, SrH and Ce) coatings on LiCoO_2_ have attracted much interest. A co-precipitation method has been utilized to prepare FePO_4_-coated LiCoO_2_ [10]. The FePO_4_ coating improves the anti-overcharge and thermal stability as well as the structural stability. Kim et al. made an extensive study on the effect of the MPO_4_ nanoparticle coatings on LiCoO_2_ cathode material [11]. They found that the extent of the coating coverage is affected by the nanoparticle size and morphology despite the same coating concentration and annealing temperature. Smaller nanoparticles of AlPO_4_ or FePO_4_ with a size less than 20 nm fully encapsulate LiCoO_2_, whereas CePO_4_ particles with a size larger than 150 nm or whisker-shaped SrHPO_4_ only partially cover LiCoO_2_. Not surprisingly, the LiCoO_2_ fully covered by AlPO_4_ or FePO_4_ exhibits the highest intercalation capacity of 230 mAh/g in a voltage range of 4.8 and 3 V at a rate of 0.1 *C*. The AlPO_4_-coated LiCoO_2_ also shows the best capacity retention. Nevertheless, the CePO_4_- and SrHPO_4_-coated cathodes show better capacity retention than the FePO_4_-coated cathode at 90 ºC, which is attributed to the continuous Fe metal ion dissolution at this temperature. The improvement in the electrochemical performance in the coated cathode is ascribed to the suppression of cobalt dissolution and the non-uniform distribution of local strain by the coating layer. In a further investigation of AlPO_4_-coated LiCoO_2_, electrochemical properties of AlPO_4_-nanoparticle-coated LiCoO_2_ at various cutoff-voltages were found to depend on the annealing temperature [12]. The AlPO_4_-coated cathodes exhibit excellent electrochemical performance with high cutoff voltages larger than 4.6 V when annealed at 600 and 700 ºC, while such cathodes annealed at 400 ºC show a lower capacity and poorer rate capability. However, the AlPO_4_-coated LiCoO_2_ annealed at 400 ºC showed optimal capacity retention [13]. Figure 2 shows typical TEM images of AlPO_4_-coated LiCoO_2_ deposited at room temperature, 400 ºC and 700 ºC. A continuous layer of AlPO_4_ with thickness of about 100 nm is coated on the surface of LiCoO_2_, as shown in Figure 2a. The coating layer deposited at room temperature is amorphous (Figure 2b). The coating deposited at 400 ºC is composed of nanocrystals with size in the range of 3-5 nm (Figure 2c), and the coating deposited at 700 ºC consists of ~20-30 nm sized nanocrystals (Figure 2d). The dependence of electrochemical properties on annealing temperature can be explained by the effect of temperature on the nanostructures of the coating layer and the interdiffusion at the interface between the coating layer and the LiCoO_2_ cathode.

In addition to coatings of phosphates, surface modification of LiCoO_2_ by coating various oxides such as ZrO_2_ [14], Al_2_O_3_ [15], SnO_2_ [16], MgO [17], ZnO [18] or 3LaAlO_3_:Al_2_O_3_ has been widely investigated. In the case of ZnO-coated LiCoO_2_, the ZnO coating reduces the cobalt dissolution and prevents the inorganic surface films such as LiF from covering the LiCoO_2_ particles [18]. Moreover, the ZnO coating alleviates the cycle-life degradation caused by inappropriate conductive carbon. Based on the impedance spectra, the charge-transfer resistance of ZnO-coated LiCoO_2_ is much smaller than the uncoated cathode, although the ZnO coating layer is more resistant than the LiCoO_2_ surfaces. It can be concluded that surface modification with ZnO improves the high-voltage cycleability of the LiCoO_2_ cathodes. In a similar manner, ZrO_2_ coating protects the LiCoO_2_ cathode surface and reduces the electrolyte decomposition at high voltages [19,20]. The ZrO_2_-coated LiCoO_2_ shows much better structural change behaviors than the bare LiCoO_2_, as evidenced by in situ XRD data. Cheng-Zhang Lu et al. have prepared LiCoO_2_ coated with different wt% of 3LaAlO_3_:Al_2_O_3_ [21]. LaAlO_3_ is used as the electrical conductor and amorphous Al_2_O_3_ help to collect PF_6_^-^, PF_5_ and HF in the electrolyte. The 3LaAlO_3_:Al_2_O_3_-coated LiCoO_2_ with 1.0 wt% 3LaAlO_3_:Al_2_O_3_ exhibits the best electrochemical performance, showing a discharge capacity of 138 mAh/g after 182 cycles which is larger than that is retained by pristine LiCoO_2_ after 38 cycles.

The battery cells discussed above all employ liquid organic electrolytes which are flammable and cause safety concerns. Replacing the liquid electrolyte with nonflammable solid electrolyte such as sulfide electrolyte is a solution to the safety problems, however, the energy densities and power densities of solid-state lithium batteries are relatively low for practical applications. One way to improve the rate capability of solid-state batteries is to add a buffer film with a thickness in nanometer scale between the electrode and electrolyte materials. A thin layer of Li_4_Ti_5_O_12_ with thickness of a few nanometers was chosen to be coated on the LiCoO_2_ cathode [22]. The Li_4_Ti_5_O_12_ is also a Li intercalation material which ensures the electronic conduction, however, this material intercalates lithium ions at voltages lower than 1.5 V and thus does not act as intercalation material in the voltage range of LiCoO_2_. The power densities of the solid-state batteries with the thin Li_4_Ti_5_O_12_ layer between the LiCoO_2_ cathode and sulfide electrolyte are greatly increased and comparable to those of commercial lithium batteries, which is attributed to the suppression of the lithium-ion transfer. A mechano-thermal method has also been used to prepare Li_4_Ti_5_O_12_-coated LiCoO_2_ particles [23]. The Li_4_Ti_5_O_12_ coating suppresses the increase of the impedance and the dissolution of Co ions. In addition to Li_4_Ti_5_O_12_-coated LiCoO_2_, LiFePO_4_-coated LiCoO_2_ has been fabricated via a impregnation method [24]. The LiFePO_4_ coating suppresses the decomposition of the LiCoO_2_ and protects the active sites as well.

LiMn_2_O_4_ is another popular cathode material for lithium-ion batteries. In comparison with LiCoO_2_ LiMn_2_O_4_ possesses essential advantages of less toxicity and having an abundant materials source. In principle, Li_*x*_Mn_2_O_4_ permits the intercalation/extraction of lithium ions in the range of 0 < *x* < 2 [25]. For intermediate values of *x* between 1 and 2 the material consists of two different phases—cubic in bulk and tetragonal at the surface. However, LiMn_2_O_4_ or substituted LiMn_2_O_4_ suffers from capacity fading especially at elevated temperatures. Coating of nanosized oxides on LiMn_2_O_4_ will help to improve its cycling performance. The electrochemical behavior of nanosized ZnO-coated LiMn_2_O_4_ was examined at 55 ºC [26]. After 50 cycles at 55ºC, the coated LiMn_2_O_4_ shows capacity retention of 97%, much higher than the capacity retention (58%) of the bare cathode. ZnO coating collects HF from the electrolyte and thus decreases the Mn dissolution in the electrolyte then subsequently reduces the interfacial resistance.

**Figure 3:**
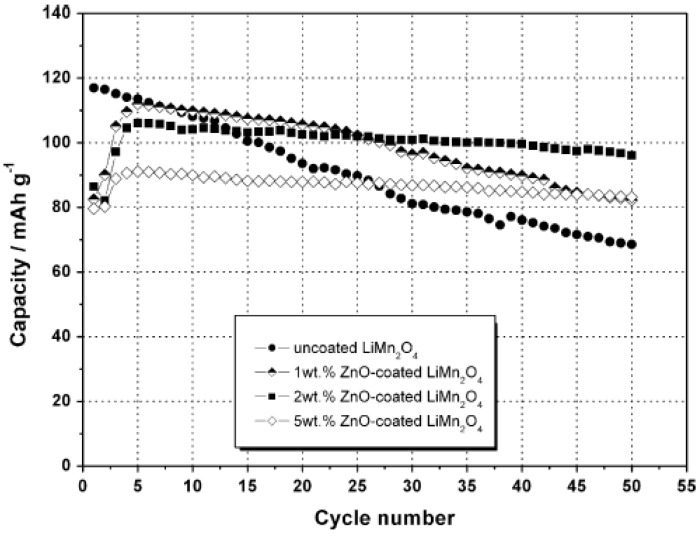
Capacity vs. cycle number profiles of uncoated and ZnO-coated LiMn_2_O_4_ cycled at 55 ºC, between 3.4 V and 4.3 V. Adapted from [27]. Copyright 2007 Elsevier.

A sol-gel route has been used to coat nanosized ZnO on LiMn_2_O_4_ and ZnO coatings with different wt% are studied [27]. Figure 3 shows the capacity *vs*. cycle number profiles of ZnO-coated and uncoated LiMn_2_O_4_ when cycled between 3.4 and 4.3 V at 55 ^o^C. The uncoated LiMn_2_O_4_ has higher initial discharge capacity but exhibits poor cycling stability, while ZnO (2 wt%)-coated LiMn_2_O_4_ shows the best cycling performance under a current rate of *C*/2. For the same reason, nanosized ZnO homogenously coated on the Li_1.05_Al_0.1_Mn_1.85_O_3.95_F_0.05_ by a hydrothermal process was found to significantly improve cycling performance of the cathode at 55 ºC [28]. The coated Li_1.05_Al_0.1_Mn_1.85_O_3.95_F_0.05_ shows high capacity retention of 98.5% after 50 cycles.

Similarly, coating of amorphous ZrO_2_ on LiMn_2_O_4_ can improve the high-temperature cycleability by picking up acidic species from electrolyte [29]. Moreover, the ZrO_2_-coated LiMn_2_O_4_ exhibits tremendously improved cycling stability at high rates up to 10*C* due to the following mechanisms. First, ZrO_2_ can form a few stable phases with Li and thus amorphous ZrO_2_ matrix possibly possesses high solubility of Li. Therefore, the ZrO_2_ coating can act as a highly-Li conducting solid electrolyte interface which reduces the interfacial resistance. Second, the rigid oxide coating strongly bonds to LiMn_2_O_4_ which tolerates the lattice stress resulted from volume expansion during lithium intercalation. Lastly, ZrO_2_ can collect HF from electrolyte to reduce Mn dissolution like ZnO does. The electrochemical behavior of ZrO_2_-coated stoichiometric LiMn_2_O_4_ and substituted Li_1.05_M_0.05_Mn_1.9_O_4_ (M = Al, Ni) cathodes were further compared with those of cathodes coated with Al_2_O_3_ and SiO_2_. ZrO_2_-coated Li_1.05_M_0.05_Mn_1.9_O_4_ (M = Al, Ni) shows the best cycling stability at 50 ºC [30]. The ZrO_2_ coating, deposited from colloidal suspensions, is porous network connected by ZrO_2_ nanoparticles with dimensions less than 4 nm. This ZrO_2_ network effectively scavenges HF from the electrolyte and allows the access of the electrolyte to the cathode, and thus improves the high-temperature cycleability of the cathode.

Li_4_Ti_5_O_12_ can also be coated on LiMn_2_O_4_, because it has a high chemical diffusion coefficient (10^-6^ cm^2^/s) [31], the same spinel structure and zero-strain property as LiMn_2_O_4_ [32]. Li_4_Ti_5_O_12_-coated LiMn_2_O_4_ particles have been synthesized by a sol-gel method [33]. The LiMn_2_O_4_ particles are enwrapped by a Li_4_Ti_5_O_12_ thin layer which is inactive to the electrolyte and does not expand during cycling. Hence, the decomposition of LiMn_2_O_4_ is hindered and the cycling stability is enhanced.

In addition to conventional lithium transition metal oxides such as LiCoO_2_, LiMn_2_O_4_, and LiNiO_2_, their derivatives have drawn tremendous attention as the cathode materials in recent years. LiNi_0.8_Co_0.2_O_2_ has both advantages of LiCoO_2_ and LiNiO_2_, and shows better structural and thermal stability than both of them. However, the safety of LiNi_0.8_Co_0.2_O_2_ needs to improve due to the poor thermal stability [34,35,36]. To solve this problem, LiNi_0.8_Co_0.2_O_2_ is coated with Al_2_O_3_ [37] and Co_3_(PO_4_)_2_ [38], respectively. The thin coating of Al_2_O_3_ (4-6 nm) separates the cathode with the liquid electrolyte, and thus reduces the side reactions. The Co_3_(PO_4_)_2_ coating can also suppress the dissolution of Ni^4+^. Moreover, the onset temperature of exothermic reactions is increased to 218 °C, which further improves the thermal stabilities. Compared to LiNi_0.8_Co_0.2_O_2_, LiNi_1/3_Co_1/3_Mn_1/3_O_2_ shows better safety performance and higher capacity (~200 mAh/g), however, its cycling stability is not satisfactory at high voltage. A combination of co-precipitation method and spray drying process is utilized to prepare the ZrO_2_-coated LiNi_1/3_Co_1/3_Mn_1/3_O_2_ [39]. ZrO_2_ scavenges the HF in the electrolyte and thus suppresses the increase of the cell resistance. Thus, the rate capacity and cycling stability are improved at a high cutoff voltage of 5 V.

### 2.2. Nanosized coatings on lithium phosphates

Lithium phosphate is presently the focus of much interest as the cathode for lithium-ion batteries, because it is inexpensive, abundantly available, environmentally friendly, thermally stable in the fully charged state and has a large theoretical capacity of 170 mAh/g. The results on the diffusion coefficient of LiFePO_4_ are controversial, because there is no compositional variation and what is measured is the movement of the LiFePO_4_/FePO_4_ interface. A diffusion coefficient around 10^-13^-10^-14^ cm^2^/s over a whole range of composition was reported by Franger *et al.* for LiFePO_4_ [40]. Another experimental work reported a value of 2 × 10^-14^ cm^2^/s [41]. Most recently, a systematic study of LiFePO_4_ with cyclic voltammetry (CV) has been presented [42]. In this study, the lithium diffusion coefficients were determined by CV to be 2.2 × 10^-14^ and 1.4 × 10^-14^ cm^2^/s for charging and discharging LiFePO_4_ electrodes in 1 M LiPF_6_ ethylene carbonate/diethyl carbonate, respectively. There are essentially no electronically conducting species in pure LiFePO_4_. Therefore, the conductivity of the material is only 10^-11^ S/cm partially due to the motion of lithium ions [43]. Carbon containing precursors (e.g. carbonates, acetates and oxalates) are used to prepare LiFePO_4_ so that some residual carbon will prevent the formation of ferric ions. The as-prepared samples show higher conductivities, in the range of 10^-5^-10^-6^ S/cm, however, it is not yet high enough for high power lithium batteries [44].

To increase the conductivity, the material could be doped as suggested by Chiang and coworkers [43]. However, doping may have deleterious impact if it occurs on the lithium sites. Conductive coatings deposited on the surface of LiFePO_4_ are usually employed to solve the conductivity issue. Most coatings are carbonaceous and deposited during the synthesis process. Among the carbon-coated LiMPO_4_ (M = Fe, Mn, Co, Ni) composites, the LiFePO_4_/C with surface carbon coating of 1.8 wt% achieves an electronic conductivity of 10^-2^ S/cm and shows the best electrochemical performance. Pioneering work on carbon coated LiFePO_4_ was carried out by Ravet *et al.* [45,46]. Sucrose was used as one carbon source [46] and was added on the initial hydrothermal samples [47] or during pyrolysis [48]. Other methods include thermal decomposition of pyrene [49], hydrothermal decomposition of ascorbic acid [50], citric acid based sol-gel processing [51], modified mechanical activation (MA) of acetylene black [52], and a spray pyrolysis assisted with planetary ball-milling [53]. Even olive oil, soybean oil, or butter can be used as the carbon source [54]. Among these methods, samples synthesized by heat-treatments at 700 °C assisted with a hydrothermal method show an increased electrical conductivity [52]. The modified MA process helps to decrease the carbon content in the cathode, and thus increase the energy density. The electrode prepared via a spray pyrolysis assisted with the planetary ball-milling shows a nearly constant discharge capacity of 114 mAh/g over 100 cycles at the 5 *C* rate, demonstrating excellent rate capacity and cycling stability.

It should be noted that the electrochemical properties of LiFePO_4_ are influenced by the quality of carbon coatings. Wilcox *et al.* found that the conductivity and rate behavior of LiFePO_4_ are strongly affected by carbon structural factors such as sp^2^/sp^3^ and disordered/grapheme (D/G), as determined by Raman spectroscopy, and H/C ratios determined from elemental analysis [55]. The structure of carbon can be controlled by the use of additives during LiFePO_4_ synthesis. LiFePO_4_ coated with the more graphitic carbon has higher conductivity and shows better electrochemical performance. Another factor that influences the electrochemical performance of LiFePO_4_/C composites is the porosity. Gaberscek *et al.* prepared microsized porous LiFePO_4_/C particles with different morphology by using different techniques such as solid-state or sol-gel methods [56]. The composite porosity is influenced by synthesis and synthesis parameters. The composites prepared at a relatively high heating rate (>5K/min) have interconnected pores and show the best electrochemical performance, e.g., more than 140 mAh/g at *C*/2 rate during continuous cycling.

In addition to carbon coating, metal coating such as silver [57] and oxide coating such as zinc oxide [58] have been successfully used to increase the conductivity as well. ZnO-coated LiFePO_4_ shows better cycling performance than bare LiFePO_4_, because ZnO coatings improve the chemical stability of LiFePO_4_ by hindering the iron dissolution. Another type of coating is conductive inorganic layer such as metallic Fe_2_P, as investigated by Rho *et al.* [59]. In their study, mixture of Fe_2_P and FeP were deposited on the surface of the LiFePO_4_ along with carbon and the by-product Li_3_PO_4_ by surface reduction reactions. Fe_2_P is coated directly on the LiFePO_4_, while carbon and Li_3_PO_4_ sit on the outer surface of the crystallites. Such surface layer structure facilitates significantly improved rate capabilities and superior cycleability: a high capacity of 105 mAh/g is achieved at a very high rate of 14.8*C*. Recently Wang and Su’s group have designed a LiFePO_4_ spherical structure coated by a π-bond character planar polymer - polyacene (PAS) - by pyrolysis of the phenol-formaldehyde resin [60]. The conductivity of LiFePO_4_-PAS structure is drastically increased to 10 S/cm. High capacities and excellent cycling performance are achieved for the LiFePO_4_-PAS structure in a wide temperature range of -20 to 60 ºC. In another study presented by Goodenough’s group, conductive polymer polypyrrole (PPy) was bonded to LiFePO_4_ particles by a carbon coat and was found to significantly improve the capacity and rate capability of LiFePO_4_ [61]. For example, at a high rate of 10*C*, the C-LiFePO_4_/PPy containing 16 wt% PPy shows a high capacity and steady cycling performance. C-LiFePO_4_/PPy composites can also been synthesized by electrochemical deposition and simultaneous chemical polymerization, in which PPy substitutes the inactive carbon and thus improves the electrochemical performance [62].

**Figure 4:**
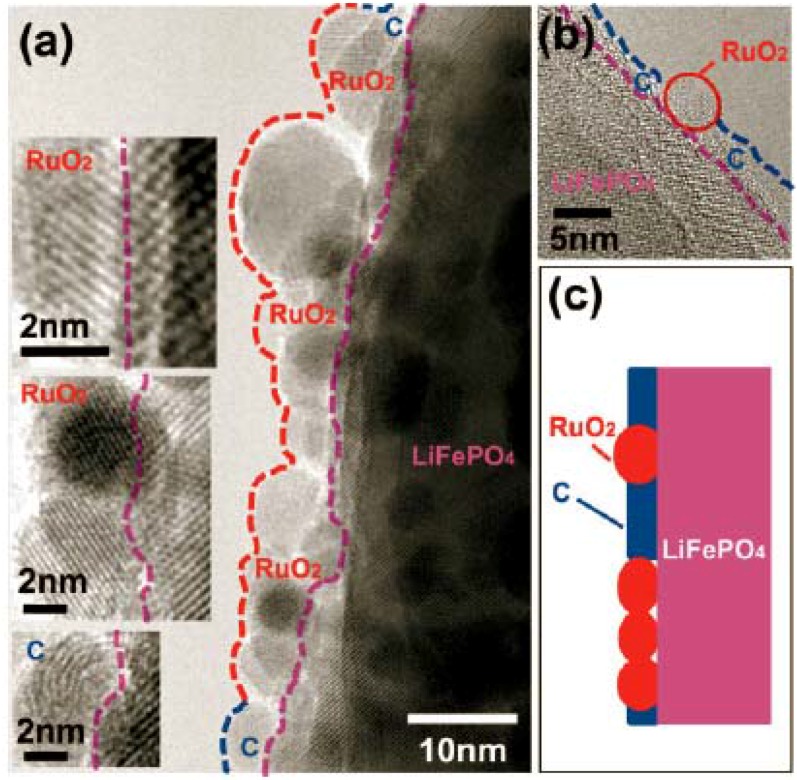
(a and b) High resolution TEM images of C-LiFePO_4_ after RuO_2_ coating. (c) Schematic of the repair of the electronically conducting network of carbon on porous LiFePO_4_ by nanosized RuO_2_. Adapted from [63]. Copyright 2007 Wiley-VCH.

In a similar manner, electronically conducting RuO_2_ was used as an oxidic nanoscale interconnect for carbon containing porous LiFePO_4_ to improve electrode performance [63]. RuO_2_ with a particle size of about 5 nm was deposited on the carbon-LiFePO_4_ with an average pore size of 50 nm by using cryogenic decomposition of RuO_4_ at low temperature. The resulting C-LiFePO_4_/RuO_2_ composite maintains the morphology and structure of the original C-LiFePO_4_, as revealed by high-resolution TEM images in Figure 4. Nanosized RuO_2_ as an oxide adheres well with oxides such as LiFePO_4_, while simultaneously assures good contact with carbon. Hence, RuO_2_ repairs incomplete carbon network in porous LiFePO_4_ and thus improves the kinetics and rate capability of the composite. It is found that the original C-LiFePO_4_ electrode shows decent performance at low current rates but the performance deteriorates at high current rates. The C-LiFePO_4_/RuO_2_ shows improved electrochemical behavior at high rates.

**Figure 5:**
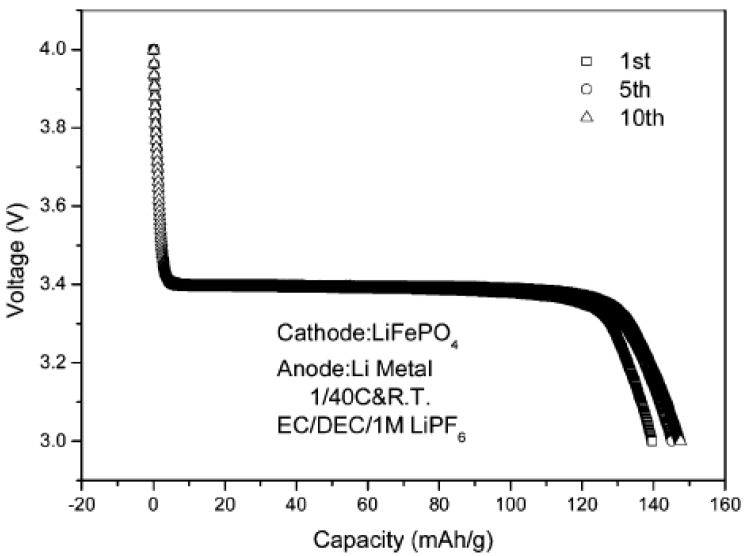
Discharge curves of LiFePO_4_/carbon sintered at 850 ºC for 2 hours. Adapted from [65]. Copyright 2004 Royal Society of Chemistry.

The problems of low electronic conductivity and slow diffusion of lithium ions in LiFePO_4_ can be further alleviated by modifying with conductive species and by minimizing particle size simultaneously, for example, a nanocomposite of LiFePO_4_ with a carbon xerogel could be formed from a resorcinol-formaldehyde precursor. This nanocomposite achieves 90% theoretical capacity at *C*/2 with very good stability at room temperature [64]. Such excellent electrochemical performance is attributed to modification with carbon and control of particle size to nanometer scale. Both factors are of essential importance. The nanosized LiFePO_4_/carbon composites with dimensions in the range of 20-30 nm could also be prepared by using citric acid as a complexing agent and a carbon source, which suppresses the growth of LiFePO_4_ particles and enhances the conductivity of the composites [65]. The carbon-coated LiFePO_4_ sintered at 850 ºC demonstrates the highest conductivity of 2.46 × 10^-3^ S/cm and best electrochemical properties, as shown in Figure 5. The discharge profile is flat over a wide voltage range, due to the two-phase redox reaction via a first-order transition between FePO_4_ and LiFePO_4_ [66]. A discharge capacity of 148 mAh/g is achieved for this cathode material. A slight increase in capacity is observed after a few cycles, showing good cycleablity.

## 3. Nanostructured Composites as Cathode Materials

Most cathode materials with interesting thermodynamic properties are typically ceramic materials with low electronic conductivity ranging from 10^-3^ S/cm for LiCoO_2_ [67] down to 10^-9^ S/cm for LiFePO_4_ [43]. To improve the electrochemical kinetics, the cathode materials need to be embedded within an electronically-conducting network, e.g., some thin coating of conductive material. The coatings must be thin enough, within nanoscale so that ions can penetrate through them without appreciable polarization. Furthermore, the internal electrical field generated by electrons may enhance the ionic motions [68]. Such surface modifications alleviate the problem of low electronic conductivity, at the same time, reducing the size of active material would shorten the diffusion length for lithium. The realization of such nanostructured composites consisting of cathode materials and conductive additives makes it possible to utilize theoretical capacities at intermediate or even higher rates.

### 3.1. Nanostructured carbon-oxide composites

One of the commonly studied carbon-based composites is carbon-vanadium oxide composite. Vanadium oxide is a typical intercalation compound as a result of its layered structure. For Li-ion intercalation applications, vanadium oxide offers the essential advantages of low cost, abundant source, easy synthesis, and high energy densities. Orthorhombic crystalline V_2_O_5_ consists of layers of VO_5_ square pyramids that share edges and corners [69,25]. The reversible electrochemical lithium intercalation into V_2_O_5_ at room temperature was first reported by Whittingham in 1975 [70]. Carbon-coated V_2_O_5_ nanoparticles can be synthesized via buring off carbon-coated V_2_O_3_ nanoparticles around 400 ºC in air [71]. The thickness and weight percentage of carbon can be manipulated by varying the conditions of the burning process. The optimal carbon content is found to be 2-3% by weight. Because of the carbon coating, these C-V_2_O_5_ nanoparticles have good interparticle electrical contact, and do not have the usual drawbacks of nanoparticles such as poor active mass integrity and high surface reactivity. Therefore, carbon-coated V_2_O_5_ nanoparticles are found to have higher capacity, better rate capability and cycleability than V_2_O_5_ microparticles or nanoparticles. The Li intercalation capacity of C-V_2_O_5_ nanoparticles reaches 290 mAh/g at high rates.

Higher capacities can be achieved with vanadium oxide/carbon nanotube nanocomposites. Dunn’s group incorporated V_2_O_5_ aerogels into single-wall carbon nanotubes using a sol-gel method [72]. The carbon nanotubes and V_2_O_5_ nanoribbons in the aerogel have similar morphology and dimensional scale, and thus have intimate contact with each other in the nanoscale. Moreover, the porous structure of carbon nanotubes and V_2_O_5_ aerogel permits electrolyte access throughout the composite material. As a result, such nanocomposite electrode shows high capacities exceeding 400 mAh/g at high rates. Akira Kuwahara *et al.* have prepared V_2_O_5_-C composites by drying the precursor which contains V_2_O_5_ sol, carbon and a surfactant [73]. The authors claim that short lithium diffusion distance (< 13 nm) and high electric conductivity (> 3.4 S/cm) are both required to attain good electrochemical performance at a high discharge rate (30 A/g).

Apart from vanadium oxides, some nanostructured lithium vanadium oxides have also been reported to form nanocomposite with carbon which exhibits excellent electrochemical characteristics. It was reported that mixing the precursor of Li_*1+α*_V_3_O_8_ with a suspension of carbon black resulted in nanocomposites of Li_*1+α+x*_V_3_O_8_/β-Li _1/3_V_2_O_5_/C [74]. β-Li_1/3_V_2_O_5_ was a by-product formed when the initial Li_*1+α*_V_3_O_8_ was reduced by carbon. Here carbon particles play critical roles as a reducing agent, a growth-limiting agent to restrict the electroactive material within the nanoscale, and as an electronically conducting agent. The Li_*1+α+x*_V_3_O_8_/β-Li_1/3_V_2_O_5_/C nanocomposite shows significantly better electrochemical performance in comparison with the standard Li_*1+α*_V_3_O_8_.

In addition to traditional nanostructured layered materials that intercalate guest species between the interlayers, there are other inorganic compounds demonstrating high lithium storage capacity by electrochemically reacting with lithium ions. For example, the lithium intercalation of nanostructured manganese oxide involves formation/decomposition of lithium oxide, which is facilitated by formation of metallic manganese. Similar to other transition metal oxides, MnO_2_ also has the problem of poor electric conductivity. Carbonaceous material can be added to MnO_2_ to improve the electronic transport. MnO_2_-CNT hybrid coaxial nanotubes have been fabricated from porous alumina template by vacuum infiltration assisted with chemical vapor deposition [75]. The MnO_2_-CNT composites exhibit a shell-core structure, in which the CNTs improve the electric conductivity considerably, supply a buffering zone, and contribute to the reversible capacity. Such nanocomposite electrode delivers a capacity higher than the unsupported MnO_2_ nanotubes by one order of magnitude. Similar to carbon black, acetylene black was also used to prompt the reduction of potassium permanganate, yielding amorphous manganese oxide/carbon composites [76]. The as-prepared composite delivers a high capacity of 231 mAh/g at a current density of 40 mA/g, showing good electrochemical performance at high rates.

The energy density of MnO_2_/C nanocomposite can be further increased by optimization of the synthesis conditions. The MnO_2_/C nanocomposite can be obtained via a sonochemical synthesis method using acetylene black and sodium permanganate. Synthesis conditions such as the reaction temperature and specific surface area of the carbon have been optimized to achieve the best electrochemical performance of the nanocomposite [77]. The active material content increases by increasing the reaction temperature. It is interesting to note that the capacity increases with the increasing amount of active material then decreases, because the excessive formation of active material increases the electrochemicaly effective volume, leading to capacity drop. On the other hand, using carbon with higher surface area results in higher capacity; the highest capacities are 126 and 99.9 mAh/g at current densities of 1 and 10 A/g, respectively.

A number of lithium phosphates/carbon composites have also been studied as cathode materials for lithium batteries, including those of general formula LiMPO_4_ (M = Fe, Mn, Co, Ni) [78] and Li_3_V_2_(PO_4_)_3_ [79]. Among these lithium transition metal phosphates with an olivine-type structure, LiFePO_4_ attracts considerable interest since 1997 [66] due to its low cost, environmental compatibility, thermal stability and relatively high theoretical capacity (170 mAh/g) [52,80,81]. Forming composite with carbonaceous material can reduce the resistance and increase the rate capacity of the electrodes. The LiFePO_4_-C nanoparticles have been prepared by solid-state reaction [82]. It is found that the discharge capacities change with different carbon source. Bo Jin *et al.* [83] have synthesized the LiFePO_4_-multiwalled CNT by a combination of hydrothermal method, ball-milling and heating. The size of particles is influenced by the multiwalled CNTs. And the electronic conductivity is further improved up to 1.08 × 10^-1^ S/cm.

**Figure 6:**
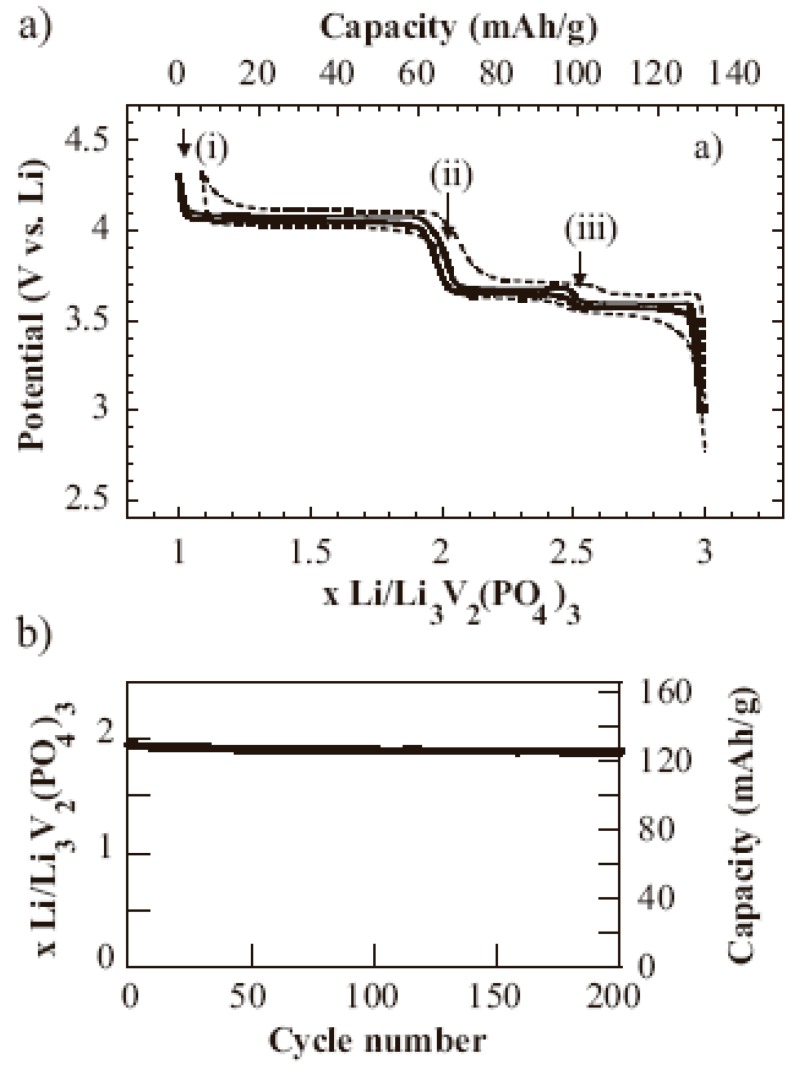
(a) Voltage-composition plot for C/Li_3_V_2_(PO_4_)_3_ composites at rates of C/5 (solid line) and 5C (dotted line) in the potential window 3.0 – 4.3 V; single phase compositions are indicated: x = 2.5 (i); 2.0 (ii); and 1.0 (iii). (b) Cycling stability at a rate of 1C. Adapted from [79]. Copyright 2002 Wiley-VCH.

Compared to LiFePO_4_, that attracts a lot of attention, Li_3_V_2_(PO_4_)_3_ is relatively unexplored. Li_3_V_2_(PO_4_) has a high operating voltage of 4.0 V and a theoretical lithium storage capacity of 197 mAh/g [79,84,85,86,87,88]. However, this material also suffers from low electronic conductivity similar to LiFePO_4_. To solve this issue, Li_3_V_2_(PO_4_)_3_ crystallites were wrapped within a conductive carbon network to form a nanocomposite which delivers almost full capacity at high rates [79]. The potential curves in Figure 6(a) reveal that two lithium ions per formula unit are completely extracted in three steps to give a theoretical capacity (100%) of 132 mAh/g at a rate of *C*/5. 95% theoretical capacity is still achieved at a high rate of 5*C*. The flat plateaus in the curve correspond to Li_*x*_V_2_(PO_4_)_3_, where *x* = 2.5 (i); 2.0 (ii); and 1.0 (iii). Such a sequence of phase transitions between two single phases shows the very low degree of polarization in the discharge curve owning to the facile ion and electron transport. Excellent cycling stability is also demonstrated by this material, as shown in Figure 6(b). When cycled between 3.0 V and 4.8 V, the Li_3_V_2_(PO_4_)_3_/C composite delivers a specific energy density of 2,330 mWh/cm^3^ comparable to LiCoO_2_ (2,750 mWh/cm^3^) or LiFePO_4_ (2065 mWh/cm^3^). Nanocarbon-coated Li_3_V_2_(PO_4_)_3_ prepared via a sol-gel process has also been reported [89]. Another promising substitute for LiCoO_2_ is Li[Ni_1/3_Mn_1/3_Co_1/3_]O_2_ due to its thermal stability [90], low cost, and low toxicity [91]. However, the electrical conductivity of Li[Ni_1/3_Mn_1/3_Co_1/3_]O_2_ is not as satisfactory as that of LiCoO_2_ [92]. Carbon coating on the Li[Ni_1/3_Mn_1/3_Co_1/3_]O_2_ is proved to improve the electrical conductivity and thus enhance the high rate capacity [64]. Citric acid [91] and super P carbon black [92] have been used as carbon sources to prepare the C-coated Li[Ni_1/3_Mn_1/3_Co_1/3_]O_2_. The resulted nanocomposite electrodes show better cycling performance, rate capability and thermal stability.

### 3.2. Nanostructured polymer-oxide composites

Over the past two decades much interest has been placed on the conductive polymer/transition metal oxide nanocomposite. The hybrid material consists of conductive organic polymers (e.g., polyacetylene, polyaniline and polypyrrole (PPy), pyridinesulfonic acid (PSA)) interleaved between the layers of an oxide lattice such as V_2_O_5_. Both oxide and polymer are electrochemically active and this feature makes the polymer/oxide nanocomposite very attractive as the cathode material for lithium batteries. A PSA-PPy-V_2_O_5_ composite has been prepared via a chemical polymerization process [93]. The addition of PSA increases the spacing between the oxides, resulting in a more homogenous hybrid material with better electrochemical characteristics. The Layer-by-Layer (LbL) technique, based on physical adsorption of oppositely charged layers, has been widely used to prepare V_2_O_5_ nanocomposites alternating with polymer layers. One popular example is V_2_O_5_/polyaniline nanocomposite film fabricated by the LbL technique and the intimate contact between the oxide and polymer within nanoscale results in an improved intercalation capacity [94]. Later, V_2_O_5_ nanocomposite alternating with blends of chitosan and poly(ethylene oxide) (PEO) was prepared using the LbL technique and investigated the charge storage capability in such nanoarchitectures [95]. A small amount of chitosan (1%) is added to blend with PEO because the adsorption of alternate layers of PEO and V_2_O_5_ is not efficient. The V_2_O_5_/blend shows higher capacity and intercalates 1.77 moles of lithium per mole of V_2_O_5_. The enhanced electrochemical performance of V_2_O_5_/blend in comparison with V_2_O_5_/chitosan is due to a larger number of electrochemically active sites and faster lithium diffusion within the host material. At 20 mV/s, the charges injected were 3.29 mC/cm^2^ and 8.02 mC/cm^2^ for V_2_O_5_/chitosan and V_2_O_5_/blend, respectively.

In a more recent report, polyaniline homogeneously distributed into V_2_O_5_/polyaniline nanocomposite was found to stabilize the capacity [96]. In this study, a reverse micelle method was used to prepare V_2_O_5_/polyaniline nanofibers which exhibit improved cycling performance compared to the V_2_O_5_ nanofibers [96]. The V_2_O_5_/polyaniline nanofibers containing 30 mol% polyaniline delivers a steady capacity of about 300 mAh/g without morphology change over 10 cycles, whereas the V_2_O_5_ nanofibers do not retain the morphology after cycling. Some V_2_O_5_/polymer nanocomposite shows lower storage capacity but better cycling stability compared to pure nanostructured V_2_O_5_ [97]. As reported by Reddy *et al.*, PVP_*x*_V_2_O_5_ (*x* = 0.5, 1) nanobelts synthesized by a hydrothermal method exhibit lower capacity but better cycleability compared with V_2_O_5_ nanobelts. The authors studied the interaction between the oxide and polymer with Fourier transformation infrared spectroscopy (FTIR) and found that the hydrogen atoms in PVP are hydrogen-bonded with the oxygen atoms of the V=O bonds of V_2_O_5_ nanobelts, which effectively shields the electrostatic interaction between V_2_O_5_ interlayer and lithium ions. As discussed above, polymers can be intercalated between the interlayers of V_2_O_5_, on the other hand, V_2_O_5_ can be interleaved within a block polymer matrix as well [98]. Mayes and coworkers used a sol-gel method to prepare continuous and amorphous V_2_O_5_ phase within the poly(oligooxythylene methacrylate) (POEM) domains of a poly(oligooxythylene methacrylate)-block-poly(butyl methacrylate) (POEM-*b*-PBMA) copolymer (70 wt% POEM) up to weight ratios of 34% V_2_O_5_ [98]. The resulted nanocomposite film is flexible and semi-transparent and the redox activity of V_2_O_5_ is preserved in such nanocomposite.

Cathode materials other than V_2_O_5_ can form nanocomposites with conductive polymer as well. Poly(ethylene oxide) (PEO) was used as an electroactive polymeric binder to mix with carbon containing Li_1.1_V_3_O_8_ [99]. The resulted composite electrode shows a capacity of 270 mAh/g at a rate of *C*/5, higher than the capacity (180 mAh/g at *C*/5 rate) of the standard electrode without PEO. Such improved electrode performance is attributed to the more efficient charge-carrier collection within the composite electrode. Among all known cathode materials, elemental sulfur is the cheapest and has the highest theoretical capacity density of 1,672 mAh/g, assuming a complete reaction to yield Li_2_S [100]. However, Li/S cells suffer from low utilization of active material, because electrochemical reaction with the interior active materials is hindered by the insulated reaction products covering the sulfur particles. Moreover, the dissolved polysulfides transfer onto the surface of the Li anode, causing lithium corrosion and poor rechargeability of Li/S cells. To overcome these two problems, nanodispersed composites with sulfur embedded in a conductive polymer matrix were designed and prepared by heating the mixture of polyacrylonitrile (PAN) and sublimed sulfur [101,102]. The composite also show excellent cycling life due to the suppressed dissolution of polysulfides into the electrolyte and thus demonstrates a great potential as cathode material for lithium batteries. Conductive polymers themselves can act as cathode material, however, they suffer from low capacities and display sloping charge-discharge curves. For example, polyryrrole (PPy) is one of the most popular conductive polymers and has a specific energy ranging from 80 to 390Wh/kg [100]. To improve its capacity, a Fe^III^/Fe^II^ redox couple is physically or chemically attached to the PPy polymer backbone [103]. The examination of the PPy/LiFePO_4_ composite electrode shows that the composite has higher specific capacity and rate capability.

### 3.3. Nanostructured metal-oxide composites and other nanocomposites

**Figure 7:**
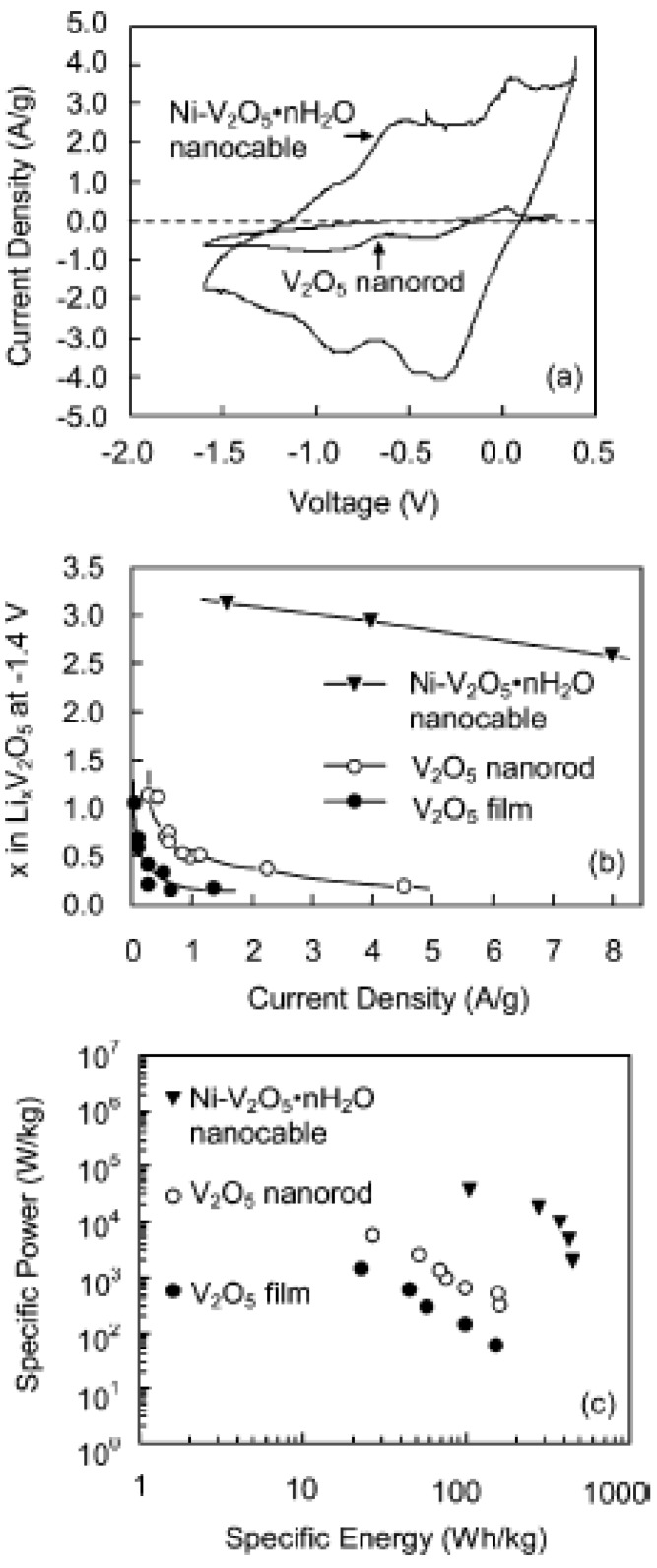
(a) Cyclic voltammograms of Ni-V_2_O_5_·nH_2_O nanocable array and V_2_O_5_ nanorod array using a scan rate of 10 mV/s. (b) Relationship between current density and Li intercalation capacity of Ni-V_2_O_5_·nH_2_O nanocable array, V_2_O_5_ nanorod array and sol-gel film from chronopotentiometric measurements. (c) Ragone plot for Ni-V_2_O_5_·nH_2_O nanocable array, V_2_O_5_ nanorod array and sol-gel film. Adapted from [104]. Copyright 2005 American Chemical Society.

The third most popular composite cathode is metal based cathode material, exemplified by the Ni-V_2_O_5_·nH_2_O core-shell structure. A two-step electrodeposition method has been used to prepare Ni-V_2_O_5_·*n*H_2_O core-shell nanocable arrays [104]. Ni nanorod arrays were first grown by the template-based electrochemical deposition. In the second step, the hydrated vanadium pentoxide shell was deposited onto the surface of nickel nanorods through sol electrophoretic deposition. Figure 7 compares the electrochemical performance of Ni-V_2_O_5_·*n*H_2_O nanocable arrays, single-crystal V_2_O_5_ nanorod arrays and sol-gel derived V_2_O_5_ films. Obviously Ni-V_2_O_5_·*n*H_2_O nanocable arrays demonstrate remarkably improved capacity and rate capability in comparison with the other two. The intercalation capacities of both nanorod arrays and sol-gel films decrease rapidly as the current density increases, while nanocable arrays are able to retain the high capacity at high current density (discharge rate), indicating the excellent high-rate performance of nanocable arrays. As shown in Figure 7c, Ni-V_2_O_5_·*n*H_2_O nanocable array has significantly higher energy density and power density than those of the nanorod array and sol-gel film by at least one order of magnitude, which is ascribed to the enhanced surface area and the reduced internal resistance.

Accordingly, Wang *et al.* synthesized Ag-Ag_0.08_V_2_O_5_·nH_2_O composite films by dispersing silver nanowires into V_2_O_5_·nH_2_O matrix [105]. The composite film is found to deliver twice the capacity of the V_2_O_5_·nH_2_O xerogel film, due to further amorphization of V_2_O_5_·nH_2_O, the increased porosity and the enhanced electronic conductivity. In a similar concept, LiCoO_2_-Ag composites have been fabricated [106]. It is suggested that Ag just enters the spacing between layers rather than substitutes the Li or Co. LiCoO_2_-Ag multilayer film was fabricated by magnetron sputtering and showed enhanced rate capability in comparison with LiCoO_2_ film of the same thickness [107]. Thickness of Ag layer is restricted within nanoscale and the rate capability of the multilayer film improves with the increased thickness of Ag layer as a result of the enhanced electronic conductivity. A citrate–nitrate combustion synthesis technique has been utilized to prepare a LiCoO_2_-Ag composite material [106]. Ag slows the decomposition in the combustion process and enhances the electrical conductivity. In the study of Rui Guo et al., a thermal decomposition method has been used to disperse Ag on the surface of Li[Ni_1/3_Co_1/3_Mn_1/3_]O_2_ [108]. The addition of Ag not only improves the electrical conductivity, but also protects the SEI film. Ag can also improve the cycling stability and high-rate discharge capacity of the LiMn_2_O_4_ electrode when dispersed on the surface using the thermal decomposition method [109].

More recently, oxide/metal/polymer composites have been obtained showing very good electrochemical performance. One example is freestanding V_2_O_5_/Pt/PVA multilayer films with the thicknesses of the V_2_O_5_, Pt, and PVA at 22, 57, and 704 nm [110]. Other types of composite structures include oxide/oxide composite such as LiFePO_4_-LiCoO_2_ and ZnO-LiFePO_4_ composites. A double-layer cathode composed of a LiCoO_2_ main layer with a LiFePO_4_ sublayer on top of Al current collector which shows better tolerance against overcharging than other electrodes including (LiCoO_2_-LiFePO_4_ mixture)/Al single layer and LiFePO_4_/LiCoO_2_/Al double layer [111]. The enhanced electrochemical performance is attributed to a large increase in the ohmic resistance of the delithiated Li_*x*_FePO_4_ layer which shuts the charging current down during overcharging without shut-down of the separator. The third type of composite structure is polymer/carbon nanocomposite, such as a polyaniline (PANI)/multiwalled carbon nanotube (CNT) composite synthesized via in situ chemical polymerization. This nanocomposite is utilized as a cathode material in a lithium metal-polymer cell assembled with ionic liquid electrolyte [112]. Such a cell demonstrates a maximum discharge capacity of 139 mAh/g with good cycleability and shows decent high rate performance (111 mAh/g at the 2.0*C* rate).

## 4. Nanostructured Composite as Anode Materials

### 4.1. Nanostructured silicon-carbon composites

Silicon is considered one of the best substitutes for carbon anodes, owning to its high theoretical capacity (4,200 mAh/g), low cost and abundant source [113,114,115]. However, bulk Si electrodes show a 400% volume variation during Li-ion insertion/extraction processes [116], which will damage the structure of electrodes and cause huge capacity loss. Dispersing Si uniformly in a host matrix proves to be an effective method to solve this problem. Carbon is not only a conductive additive, but also supplies a ductile host matrix for dispersing Si nanoparticles [117]. In addition, carbon contributes to the capacity since Li can be intercalated into carbon as well [118]. As a result, the Si-C composite shows higher capacity than bare carbonaceous materials and better cycling stability than unsupported Si electrodes [119]. A composite material composed of nano-Si dispersed in carbon was reported as early as in 1995 [120]. Jie Shu *et al.* have synthesized a cage-like CNTs/Si composite using a chemical vapor deposition method [121]. In this structure, the Si particles are wrapped by a cage formed from tortile carbon nanotubes (CNTs). The CNTs improve the conductivity and are covered by a solid electrolyte interphase (SEI) film which improves the cycling stability of the electrode. The Si-C composite can also be prepared by dispersing nanocrystalline Si into the carbon aerogel [119]. This type of Si-C composite delivers a discharge capacity of about 2,000 mAh/g at the *C*/10 rate in the first cycle and a nearly constant capacity of 1,450 mAh/g after 50 cycles.

Other than the relatively simple Si-C composites that have demonstrated their capability in improving the performance of anodes as discussed above, there are other composites with complex structures which can be used to increase the capacity and enhance the cycling stability of anodes as well. A ball milling method has been employed to prepare a composite anode material of silicon/graphite/CNTs [118]. This preparation method of ball milling generates structural defects in multi-walled carbon nanotubes (MWNTs) and leads to MWNTs with shorter length. However, it is interesting to note that the structural defects benefit the Li-ion storage and the shorter length of MWNTs facilitates the Li-ion diffusion [122]. MWNTs are added into the silicon/graphite composite due to their remarkable resiliency [123,124] and good electric conductivity [125]. Moreover, the network of MWNTs can enwrap the flaked graphite particles tightly, which further reduces the effect of the volume variation during Li-ion insertion/extraction process. Other composites with complex structures include a carbon-coated nano-Si dispersed oxide/graphite composite material prepared by Heon-Young Lee *et al.* [126]. In this structure, the graphite and inactive oxide are mainly used as the elastic matrix; the carbon-coating suppresses the side reactions between the electrolyte and the surface graphite particles and enhances the electrical contact of the Si and the graphite.

### 4.2. Nanostructured tin-carbon composites

Apart from the Si-C composites, the Sn-C composites also show excellent electrochemical performances as anode materials. Sn has a lower active voltage (0.3 V) than that of Si (>0.5 V) [127] and has a very large theoretical capacity (994 mAh/g) as one mole of Sn can store 4.4 moles of Li [128]. However, the drastic volume variation (>300%) during Li-ion insertion/extraction process still limits its practical applications [129].

The cycling stability of Sn can be improved by dispersing Sn into a carbon matrix which functions as a buffer. Bingkun Guo *et al.* have recently prepared the Sn-C composite by embedding Sn nanoparticles into the mesopores of hard carbon spherules [130]. The initial coulombic efficiency (charge capacity divided by discharge capacity) is increased remarkably due to the SEI film induced by nano-Sn. In the study of Gaelle Derrien *et a*l., Sn nanoparticles are dispersed in a carbon matrix by infiltrating a tin precursor into an organic gel followed by calcinations [128]. Volume contraction occurs in the calcinations process and creates open space in the structure, which helps to alleviate the effect of volume change during Li-ion insertion/extraction process.

**Figure 8:**
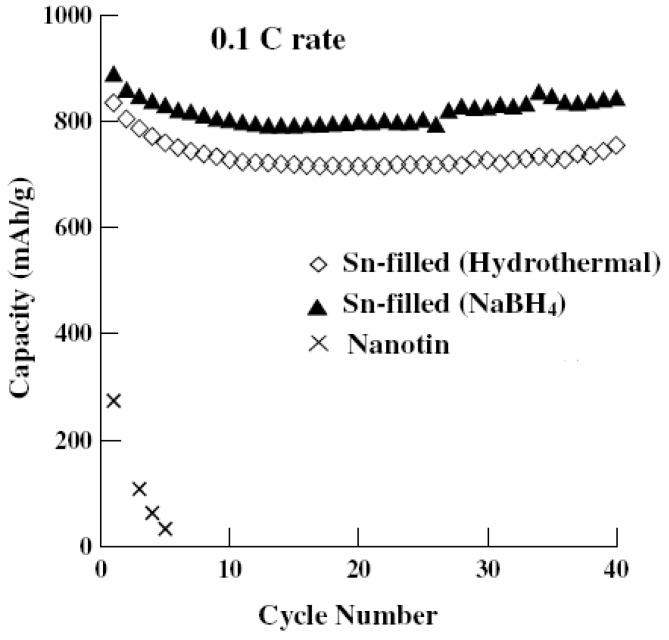
Cycling performances of nanotin and tin-filled carbon nanotubes. Adapted from [135]. Copyright 2006 Elsevier.

Carbon nanotube (CNT) has a capacity of more than 1,400 mAh/g as the anode material, but its practical application is limited due to the poor cycling stability [131,132,133,134,135]. T. Prem Kumar *et al.* have reported their synthesis of tin-filled carbon nanotubes via a hydrothermal reduction process and a NaBH4-reduction process [135]. Figure 8 shows the cycling performances of nanotin and tin-filled carbon nanotubes. The overall capacity and the cycling stability of tin-filled carbon nanotubes are improved compared to unsupported Sn and MWNT. Such nanocomposite electrode exhibits a capacity of 1,082 mAh/g for the hydrothermally reduced product and 1,585 mAh/g for the NaBH_4_-reduced product at the 0.1*C* rate. The capacities of the two products retain 754 and 844 mAh/g over 40 cycles, respectively.

Coating Sn with carbon is another method to prepare the Sn-C composite. Carbon-coated Sn nanoparticles have been synthesized via a hydrothermal method [136], or by mechanical mixing and heating the precursor subsequently [137]. MgO is added in the precursor to hinder the agglomeration of molten Sn and to provide free space for Sn after the MgO is dissolved.

### 4.3. Nanostructured tin oxide-carbon composites

SnO_2_ is a wide-band-gap (E_g_ = 3.6 V) n-type semiconductor which finds wide applications [138,139,140]. SnO_2_ is an attractive anode material because SnO_2_ can intercalate twice Li (781 mAh/g) compared to graphite [140,141,142]. However, the tin oxide anodes face the same problem of drastic volume change during cycling as the metallic tin electrodes [140,143]. The electrochemical performance of tin oxides can also be improved by forming composites with carbonaceous materials. The carbonaceous material serves as the buffering matrix to hinder the agglomeration of nanoparticles and enhance the electrical contact; it also delivers additional capacity at the same time [144,145,146,147,148]. In the work reported by Ning Du *et al.*, polocrystalline SnO_2_ naotubes have grown on the CNTs layer by layer [149]. These nanotube electrodes exhibit large reversible capacities due to the high specific surface area. SnO_2_ can also be dispersed into multi-walled CNTs by using a one-step thioglycolic-acid-assisted wet chemical method [150] or through the oxidation process in supercritical carbon dioxide-methanol solution [151]. The thickness of SnO_2_ coating can be controlled by changing pH value and hydrolysis time [150]. Hui Qiao *et al.* [152] and Yi-Chun Chen *et al.* [127] have both reported SnO_2_-C composites with core-shell structures prepared by using a one-pot solvothermal method and a sol-gel method, respectively. The solvothermal method produces the SnO_2_-C core-shell structure, and the sol-gel method yields the C-SnO_2_ core-shell structure. Other SnO_2_-C composites include SnO_2_-C hollow spheres [153] and C-coated SnO_2_ nanoparticles [154]. The C-coated SnO_2_ nanoparticles deliver a discharge capacity of ~500 mAh/g over 20 cycles. Other than SnO_2_-C composites, SnO-CNT composites have attracted some research interests and can be obtained via the sol-gel method [155]. The well-dispersed SnO can hinder or reduce the formation of the SEI film, which reduces the large capacity loss of pristine CNTs in the first cycle.

### 4.4. Nanostructured transition metal oxide-carbon composites

Apart from tin oxides, nanostructured transistion-metal oxides such as TiO_2_, CuO, NiO, and Co_3_O_4_ can also be good candidates for anode materials [156]. These nanostructured oxide electrodes all deliver higher capacities than graphite-based anodes [2]. A variety of transition metal oxides can also form composites with carbonaceous materials. Here carbon is mainly used to improve the electric contact of the electrodes, in addition to acting as a buffering matrix for dispersing host materials.

Titanium oxide is a good host for lithium ions due to its high reversible capacity, low cost, and nontoxicity [157] Another advantage offered by titanium oxide is higher operating voltage (~1.7 V vs. Li^+^ (1M)/Li) than that of carbonaceous materials (~0.1 V vs. Li^+^ (1M)/Li) [158]. TiO_2_ provides facile diffusion paths for Li ions [159], but its electric conductivity is relatively low (~10^-12^ S/cm) [160], which leads to poor cycling stability [161]. A possible method to solve this problem is to combine TiO_2_ with carbonaceous materials. The TiO_2_-carbon anodes demonstrate enhanced kinetics of lithiation/delithiation processes and increased diffusion coefficient of Li ions [159]. In the structure of TiO_2_-C core-shell composites synthesized by L.J. Fu *et al.*, the carbon shells enwrap the TiO_2_ cores and hinder the agglomeration of nanoparticles [159]. A hydrothermal method followed by calcination [160] has been used to fabricate a composite of TiO_2_ nanotubes and carbon in which nano-sized C is dispersed in TiO_2_. This composite is also a porous material with large surface area. The dispersed nano-sized carbon helps to reduce the bulk resistance, SEI resistance and charge transfer resistance in the anode. This composite material is able to maintain a discharge capacity of ~138 mAh/g over 70 cycles. Nanoporous TiO_2_-C composite can also be prepared by using a template method [161]. The conductive carbon network is coated onto the TiO_2_ in this structure, which facilitates the transportation of Li ions and electrons.

**Figure 9:**
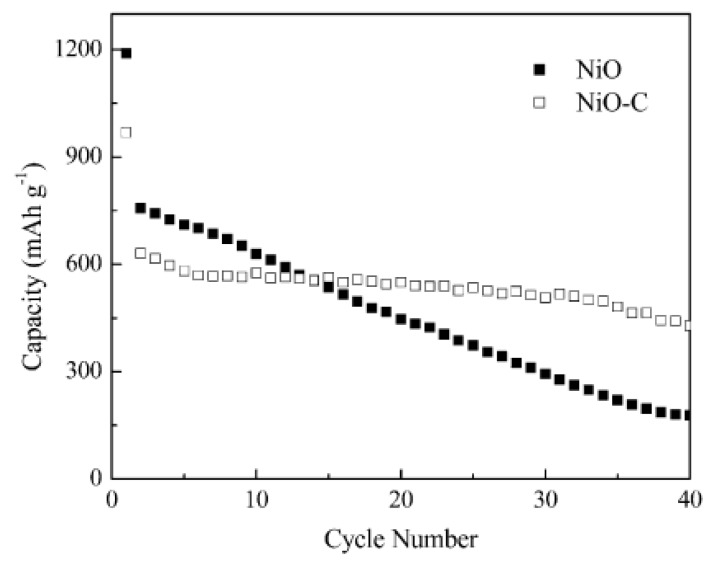
capacity vs. cycle number profiles of the NiO and NiO-C electrodes cycled between 0.02 and 3.0 V at 0.1 *C* rate. Adapted from [162]. Copyright 2007 the Electrochemical Society.

Apart from TiO_2_, NiO has a theoretical capacity of 718 mAh/g. However, NiO suffers from aggregation, pulverization and poor conductivity [162]. Tu’s group have synthesized NiO-C composites by carbonizing the net-structured [162] and spherical [163] NiO under hydrothermal condition. Carbon filled in the porous NiO stabilizes the structure and improves the electrical conductivity of NiO matrix. Figure 9 shows cycling performances of net-structured NiO and NiO-C nanocomposite electrodes at a current rate of 0.1 *C*. The composite electrodes retain a capacity of 429 mAh/g compared to that of 178 mAh/g for the NiO electrode over 40 cycles. Similar to NiO, CoO has a theoretical capacity of 715 mAh/g and the theoretical capacity of Co_3_O_4_ is 1100 mAh/g [156,164]. Nanostructured cobalt oxides containing CoO and Co_3_O_4_ can form composites with mesoporous carbon spheres [165]. The porous carbon provides good electric conductivity and buffer space, in addition to hindering the aggregation of the nanoparicles. Such an electrode delivers a capacity of 550 mAh/g after 30 cycles.

### 4.5. Other nanostructured composites

Nano-sized SnSb anodes also show high Li-ion intercalation capacities [117,166,167,168]. However, the nanoparticles with diameters less than 100 nm easily aggregate together, losing their advantage as nanomaterials and showing gradually decreased capacities due to the drastic volume change [167,168]. Xuejie Huang’s group has dispersed SnSb nanopaticles on the rigid carbonaceous spherical material such as mesophase carbon microbeads (MCMB) and hard carbon spherules (HCS) to separate these nanopaticles [169,170,171]. The SnSb content should not exceed 30 wt% in the SnSb-MCMB composite, otherwise severe aggregation will appear again. The SnSb-HCS electrode shows a high coulombic efficiency (82%) in the first cycle, which is ascribed to the less surface area exposed to the electrolyte and thus less formation of the SEI film. The SnSb-HCS electrode shows a capacity of 460 mAh/g over 35 cycles at a current density of 0.2 mA/cm^2^.

**Figure 10:**
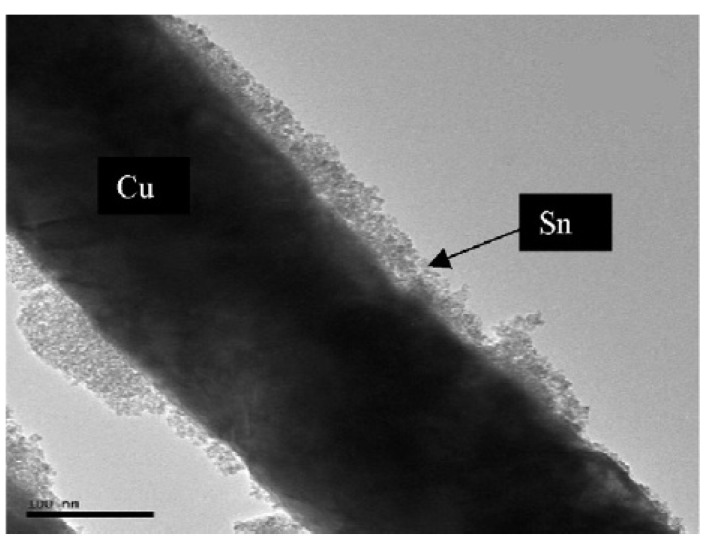
TEM image of a single Sn-covered Cu nanopillar (5 min tin deposition). Adapted from [176]. Copyright 2009 Elsevier.

In addition to carbonaceous materials, materials that are electrochemically inert to Li can also serve as a ductile host matrix for Si [172,173]. TiN is not reactive with Li in the potential range of 0.02-1.2 V in terms of electrochemistry and is not reactive with Li and Si in terms of chemistry. It also has superior electrical conductivity and mechanical strength. The optimum milling time is 12 h in this research work. The electrodes with a milling time of 6 h show poor cycling stabilities due to poor binding between the two components [117]. On the other hand, milling with longer time (18 h) may generate inactive Si, and thus decreases the capacity of the electrode. Another alloy, nanocrystalline NiSi alloy, has been reported [172] to form the active/inactive composites, in which Si acts as the active center and Ni functions as the buffering matrix. Such an anode shows a discharge capacity of 1,180 mAh/g in the first cycle and retains 800 mAh/g after 25 cycles. Ni dispersed in Co_3_O_4_ can improve the initial coulombic efficiency. Mechanical milling has been used to synthesize Ni-Co_3_O_4_ composite [174]. The initial coulombic efficiency is enhanced from 69% for bare Co_3_O_4_ to 79%, which is ascribed to the optimum contact area of Co_3_O_4_ and Ni. Si-coated Cu nanopillars are another composites composed of the active component (Sn) and inactive component (Cu) and can be fabricated by a simple electroplating method [175]. This brush-like structure with large surface area can effectively alleviate the effect of volume change during cycling and improve the power rate performance. Figure 10 shows the TEM image of one “bristle” on the “brush” and the deposition time is 5 minutes. It is clear that a tin layer with the thickness in the range of 20-50 nm is deposited on the Cu pillar.

## 5. Concluding Remarks

This review clearly reveals how moving from pristine materials to nanocomposite materials can significantly improve device performances for energy conversion and storage. The development of high-performance lithium-ion batteries can benefit from distinct properties of nanocomposites, such as excellent electric conductivity and freedom for volume change during Li-ion insertion/extraction process, as well as enhanced electrochemical, thermal and structural stability during charging/discharging cycles.

Applications of nanotechnology in energy storage are in the stage of research and development. For realization of wide industrial applications, further work is required to achieve controlled and large-scale synthesis of nanocomposite materials, to understand mechanisms of lithium storage in nanocomposite electrodes and kinetic transport on the interface between electrode and electrolyte. The effects of nanostructures in battery performance are not only simple consequences of a reduction in size or combination of two materials. Interfacial properties are subtle and critical. This challenges researchers worldwide to carry out systematic experimental studies and to develop predictive theoretical tools for better fundamental understanding of relationships between nanostructures and electrochemical characteristics of electrode materials.
